# Professional Jealousy

**Published:** 1848-04

**Authors:** J. Allen


### PROFESSIONAL JEALOUSY.

BY J. ALLEN.

As this is one of the causes which serve to keep down the dental profession, the writer deems it proper to offer a few remarks upon that subject. In order to elevate the standard of our profession, it is important that each of its members should contribute his due proportion of aid in the accomplishment of that object. This he can do by severing every tie that holds it down, so far as he is himself concerned, and by cooperating with, and sustaining other members of the profession who are entitled to confidence, not only in all the general movements that are made to raise the standard, but also to sustain his fellow practitioners in their private practice, so far as he can consistently, in an open, frank, and candid manner.

Let us look at the course pursued by many of our professional brethren in this respect. They make it a point invariably to disapprove and condemn every operation they may chance to meet with that has been performed by another; whatever merit it may possess, if not done by themselves, is entitled to no credit; they will find some fault, some hook to catch upon, by means of which they will endeavor to sink other operators as low as possible in the estimation of their patients. If an individual has had services rendered by different dentists, he will find that each one will condemn in turn all that has been done by his predecessors, the last operator endeavors to impress it upon the mind of his patient that his former operations have all been worthless, and that those who performed them were mere quacks who know nothing of the business. But, says the patient, they were highly recommended to me, and some of their work has rendered me essential service, here is a tooth I had filled twelve years ago, and there is one that has been filled seven years, and they seem perfectly good yet. Very true, replies the doctor, they may seem to be good, but you are not aware of the decay that is going on under those fillings, they are not filled as I should have filled them, if they had been, they would have lasted you forty years. Well, replies the patient, I do not wish any thing done this morning, perhaps I will call again. Thus we see that the doctor's efforts to pull down his competitors, served only to lower himself in the estimation of his applicant, for he takes his leave fully impressed with the belief that this wiseacre has missed his mark, for he savors as strongly of quackery as those he has attempted to revile.

There is a class of patients who, from their incredulity and fault finding dispositions, find ready favor with these would be wise dentists, they will complain of every dental operation they have ever had performed, whether good or bad. There is Dr. A., says one, who ruined my teeth, they were all perfectly sound except some little black spots between these front ones, which he filed, and immediately thereafter every tooth in my mouth was in a state of decay: Oh! he has ruined me. The doctor, instead of explaining the matter in an honest manner, by candidly telling the patient that according to his own admission they had commenced decaying before Dr. A. touched them, and that his filing the front teeth could not have caused the back ones to decay, thereby defending a professional brother from censure, chimes in at once with a tirade of denunciations against Dr. A. for performing perhaps the same kind of an operation that he himself had performed not an hour before, and may again upon the next patient that enters his office, without any conscientious scruples whatever. Now, if it was wrong for Dr. A. to perform a certain operation, it was equally wrong for Dr. B. to perform the same kind of one in like manner. But professional jealousy does not recognize any true principle of right. B inserts a set of teeth; C stamps his seal of condemnation upon the work. C attempts to extract a tooth and breaks it off, the patient seeks and finds condolence at another office, (Dr. D's,) who thinks it a horrid piece of work. Had you come to me, says Dr. D, before that quack got hold of it, I could have taken it out without any difficulty; but now it is doubtful whether it can ever be removed. Thus each one is profuse in his denunciations against all others of the same profession, and equally so in sounding his own praise, for these two opposites always go hand in hand, and in this way many members are more active in injuring the profession than they are in sustaining it.

With such dead weights in our midst, how can we expect our profession to assume that lofty tone of character to which it is justly entitled; and whilst so many are trying to build themselves up by putting others down. Why should this be? Does any one think that by so doing he will increase his own business? if so, he is mistaken. Should we be like a set of dogs fighting for a bone, when there is meat enough and to spare for all who are worthy. There is not one-half the dental business done yet that ought to be, nor will there be until public confidence can be secured, and this cannot be done while there are so many whose constant aim is to destroy it, and whose withering influence is ever blighting our fondest hopes.

It is an old saying that in union there is strength—in order, therefore, to raise the platform upon which our profession stands, let us put forth our united efforts, let us go harmoniously onward and upward in our professional career, mutually receiving and imparting that which is useful, and gathering gems from each other to place in our own diadems; approving and commending all that is right, and condemning only that which is founded in error.

"Oh, *Jealousy!* thou *bane* of social joy;
Oh! she's a *monster* made of *contradictions*.
Let *truth*, in all her native *charms* appear,
And with the voice of *harmony itself*
Plead the just cause of *innocence* traduced.
*Jealousy* is like
A polished *glass*, held to the lips when life's in doubt,
If there be *breath* 'twill catch the damp and *show* it.
It is its peculiar *nature*
To swell *small* things to great; nay, out of *naught*
To conjure *much*, and then to lose its *reason*
Amid the hideous *phantoms* it has formed."

